# Effectiveness of Mediterranean Diet’s Adherence in Children with Inflammatory Bowel Diseases

**DOI:** 10.3390/nu12103206

**Published:** 2020-10-20

**Authors:** Caterina Strisciuglio, Sabrina Cenni, Maria Rosaria Serra, Pasquale Dolce, Massimo Martinelli, Annamaria Staiano, Erasmo Miele

**Affiliations:** 1 Department of Woman, Child and General and Specialized Surgery, University of the Campania Luigi Vanvitelli, 81100 Naples, Italy; caterinastrisciuglio@hotmail.it; 2 Department of Translational Medical Science, Section of Pediatrics, University of Naples Federico II, 80131 Naples, Italy; sabrinacenni3@gmail.com (S.C.); mrserra.studio@gmail.com (M.R.S.); martinellimassimo@hotmail.com (M.M.); erasmo.miele@unina.it (E.M.); 3 Department of Public Health, University of Naples Federico II, 80131 Naples, Italy; pasquale.dolce@unina.it

**Keywords:** Inflammatory Bowel Disease, nutrition, Paediatric gastroenterology, Mediterranean Diet, intestinal inflammation, dietary intake

## Abstract

Background: Nutritional support is very important in the treatment of Paediatric Inflammatory Bowel Disease (IBD). The role of the Mediterranean Diet (MD) has been understudied in children with IBD. The aims of this study were to assess the dietary intakes of IBD children in comparison with healthy controls (HCs), their adherence to MD; and the relationship between inflammation and dietary behaviors. Methods: Paediatric IBD patients in clinical remission and HCs were enrolled. The nutritional status and adherence to the Mediterranean Diet was evaluated through a 3-day food diary and the Mediterranean Diet Quality Index for Children and Adolescents (KIDMED). Results: The analysis of food diaries showed a significantly higher kilocalorie intake in IBD patients compared to HCs (*p* = 0.012), an increase in carbohydrates (*p* = 0.015) and in protein intake (*p* = 0.024). Both IBD and HCs have an intermediate adherence to MD. The comparison between Crohn’s disease (CD) and Ulcerative colitis (UC) patients showed significant difference in protein intake in CD patients (*p* = 0.047), as well as for vitamin D (*p* = 0.044) and iron intake (*p* = 0.023). Interestingly; in IBD patients we found a significant association between adherence to MD and a low level of fecal calprotectin (*p* = 0.027). Conclusion: Children with IBD in remission have a sub-optimal food intake compared to HCs. MD seems to correlate to decreased intestinal inflammation.

## 1. Introduction

In Inflammatory Bowel Disease (IBD), nutritional problems are common and depend on the location, disease activity, surgery and associated complications. Between 20 and 85% of IBD patients have nutritional deficiencies, and especially protein-caloric malnutrition [1,2]. Some patients believe that food induces or may exacerbate some symptoms, and for this reason they modify their diet to gain some control over the disease. This attitude can however have an important impact on the nutritional status [3]. Indeed, families of children with IBD pay close attention to the patient’s diet and they modify it by eliminating certain foods to control gastrointestinal symptoms such as diarrhea and abdominal pain. The most commonly excluded foods are cereals (29%), milk (28%), vegetables (18%) and fruit (11%) [4]. However, several of these foods are the main components of the Mediterranean Diet (MD), which is a nutritional model inspired by the food models common in some Mediterranean countries. In recent years, this diet has been universally proposed as protective for health [5]. On one hand, avoiding certain foods without balancing the diet can inadvertently reduce the overall amount of calories, macronutrients and micronutrients taken, contributing to malnutrition and specific nutritional deficiencies in pediatric patients with IBD [6,7]. On the other hand, the latest ESPGHAN guidelines show that the patient with IBD must not be subject to particular food restrictions and that an individual diet must be assessed on the basis of the nutritional imbalances of the individual patient in order to avoid aggravating the symptoms [8,9].

Therefore, a growing body of evidence supports the need for special attention to nutrition and diet in children with IBD, although data on the diet of children with IBD is scarce. There are only few studies evaluating dietary intake in pediatric IBD, which report a limited number of patients [10,11] or lack comparison to a valid reference population [12,13]. Moreover, most of the studies analyze patients with active disease, which has been reported to alter normal nutrition habits [14].

Indeed, some evidence supports dietary behaviors as a mechanism in the relationship between active disease and nutritional status, specifically that active disease is associated with inadequate or lower caloric and micronutrient intake, whereas the relationship between chronic inflammation and altered dietary intake has not been consistent [10,11,15]. Finally, a Mediterranean dietary pattern has shown positive effects in chronic disease [16], although it has been understudied in IBD, especially in children.

Therefore, the aims of the present study were to assess the dietary intakes and anthropometrics parameters of IBD children in remission in comparison to healthy controls subjects, their adherence to MD and the relationship between biomedical factors (e.g., disease activity, inflammatory markers) and dietary behaviors. 

## 2. Materials and Methods

### 2.1. Subjects

A single center cross sectional study was undertaken at the Department of Translational and Medical Science, Section of Paediatrics, University of Naples “Federico II” from September 2018 to October 2019. Our study population included subjects aged less than 18 years with a diagnosis of Crohn’s Disease (CD) or ulcerative colitis (UC) according to the revised Porto criteria [17].

To be eligible for the study, patients were required to: (a) have a documented diagnosis of UC or CD based on clinical, radiological and endoscopic criteria; (b) be in clinical remission within three months of recruitment; (c) be in steroid-free remission for three months prior to study entry. Patients were excluded if: (a) they used corticosteroids within the preceding three months, (b) they had bowel resection or a stomy was present, (c) they used prebiotic or probiotic fiber supplements in the past three months, (d) comorbidities known to affected the nutritional status and/or bone metabolism, growth, or pubertal development, (e) they were on special diets. The baseline patients’ characteristics were extracted from patients’ medical records: age, sex, time since diagnosis, IBD phenotype according to the Paris classification [18], current medication. In IBD patients, fecal calprotectin (FC) was determined through ELISA test and values expressed in microgram per gram of feces (μgr/gr feces).

Healthy children (HCs), matched with patients for age and gender, were enrolled as control group. To obtain healthy controls, we surveyed subjects aged 4 to 18 years enrolled in schools (primary schools and secondary schools) in the same period. For each city, the schools were selected randomly from a list of all the schools available in the region.

The Principal Investigator contacted the directors of the selected schools to obtain permission to conduct the study. From each school, randomly selected classes were surveyed. Informed consent was obtained by parents/legal guardians of the involved children. 

Control subjects were excluded if they had any gastrointestinal disease or a medical condition requiring a specialized or restricted diet.

### 2.2. Anthropometric Data

Physical examination was performed at the time of recruitment in IBD children. All measurements were performed by a single investigator on the same scale and stadiometer. Weight, height and BMI, were expressed as z-scores (Anthropometric software program, Centers for Disease Control, Atlanta, GA, USA) calculated considering the general Italian population as a reference, in order to compare parameters from subjects with different age and gender, weight, height, BMI, and growth velocity z-scores. Weight was measured to the nearest 0.1 kg using a platform beam scale and height to the nearest 0.5 cm using a stadiometer. BMI was calculated as weight (in kilograms) divided by height (in meters squared) and expressed as a z-score.

### 2.3. Dietary Intake

The dietary intake was assessed using a 3-day food diary record. The patients were asked to record everything they ate and drank immediately after consumption, including the amounts consumed (in household measures), the method of cooking, and the brand names of any product used. We evaluated 2 days of the week and one day of the weekend to analyze any changes in the diet. Data from the diaries were analyzed for energy and macro and micronutrients intake, using nutrient analysis software Winfood^®^ PRO 3.9.x (Medimatica Surl, Teramo, Italy). The nutrients intake of patients with IBD was compared with recommended daily allowances (RDA) for energy, macro, and micronutrients. Moreover, dietary intake of children with IBD was compared with the control group. Adherence to the Mediterranean Diet was evaluated using a specific questionnaire, the KIDMED based on a test of 16 questions with scores ranging from 0 to 12 (>8 optimal; 4–7 intermediate, ≤3 very low adherence).

### 2.4. Ethical Considerations

The Institutional Review Board of the University of Naples “Federico II” approved the study protocol with the registration number 127/19. Written informed consent was obtained from parents, and patients older than 13 years signed a statement of assent.

### 2.5. Statistical Analysis

Data were presented as the number of patients (%) for categorical variables and as mean ± standard error of mean (SEM) or median (range), as appropriate, for quantitative variables. Comparison between groups were performed using χ^2^ test or Fisher’s Exact test for categorical variables, while Student’s *t* test or Mann-Whitney test, were used for quantitative variables, as appropriate. All comparisons between CD and UC were adjusted for sex using classical multiple linear regression. 

As for the tests for differences between patients with IBD and the reference intakes from RDA of several dietary components, the one sample Wilcoxon test was used when the reference value was a median, while the one sample Student’s *t*-test was used when the reference value was a mean.

All statistical analyses were performed with the R statistical software (version 3.4.4). Statistical significance was set as a *p*-value of <0.05. 

## 3. Results

### 3.1. Subjects

One hundred twenty five children with IBD were enrolled between September 2018 and October 2019. In the same period, we enrolled 125 healthy control subjects. Fifty three (42.4%) subjects with CD, while 72/125 (57.6%) with UC. Sixty eight (54.4%) were male and 57 (45.6%) female.

There were no significant differences in z-scores for weight and height between the IBD and HC group. Baseline clinical details of patients enrolled are summarized in Table 1.

**Figure 1:**
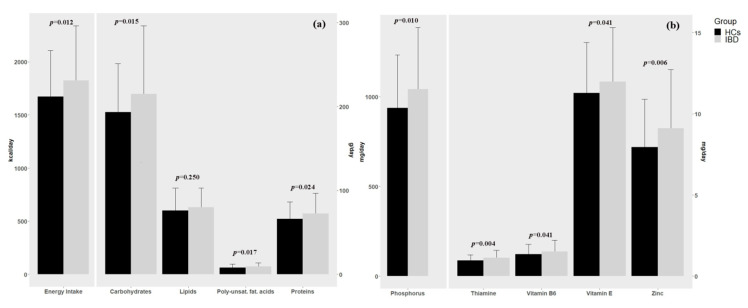
(**a**) Difference in energy intake and macronutrients between children with inflammatory bowel disease (IBD) and the Healthy controls (HCs). (**b**) Difference in micronutrients between children with IBD and the Healthy controls (HCs).

The analysis of food diaries showed a significantly higher kilocalories intake in patients with IBD compared to healthy controls (1.825.25 ± 46.12 kcal/day vs. 1673.15 ± 38.74 kcal/day, respectively, *p* = 0.012), and a significant increase in carbohydrate intake (215.00 ± 7.29 g/day vs. 193.17 ± 5.19 g/day, *p* = 0.015) and in protein intake (72.52 ± 2.17 g/day vs. 66.09 ± 1.82 g/day, *p* = 0.024). (Figure 1a and Table 2) Patients with IBD also had a significantly increased intake of several micronutrients and vitamins. (Figure 1b and Table 2; Table S1) Moreover, in the IBD group the score obtained by the KIDMED showed a level of adherence to MD optimal in 16.1%, intermediate in 56.9%, and low in 26.6%; instead in HCs group we found a level of adherence to MD optimal in 15.6%, intermediate in 61.5%, and low in 22.9% (Table 2).

### 3.2. Comparison between CD and UC

We found a significant difference in sex between the two groups with 41/72 female (56.9%) in the UC group and 16/52 (30.2%) in the CD group (*p* = 0.0037), whereas there were no significant differences in z-scores for weight and height. The analysis of food diaries adjusted for sex showed no differences in kilocalories, carbohydrates and lipids intake in patients with CD compared to UC, but a significant increase in protein intake in CD children (80.10 ± 3.20 g/day vs. 66.93 ± 2.78 g/day, *p* = 0.047). We also found significant differences in Vitamin D (10.80 ± 2.96 mcg/die in CD vs. 3.32 ± 0.92 mcg/die in UC, *p* = 0.044) and iron intake (10.52 ± 0.49 mg/die vs. 8.54 ± 0.37, *p* = 0.023) between the two groups (Table 3; Table S2).

### 3.3. Association between Dietary Habits and Markers of Diseaseactivity

**Figure 2:**
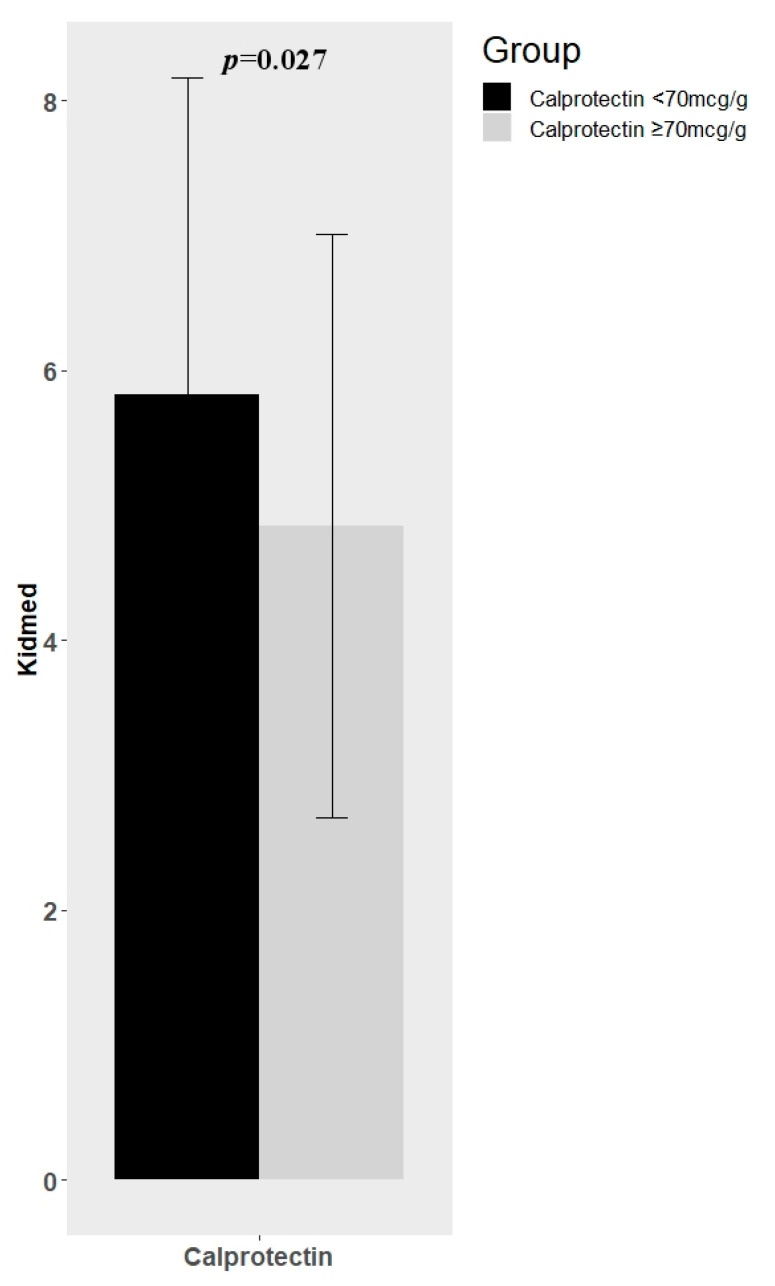
Difference in Mediterranean Diet Quality Index (KIDMED) mean between IBD (Inflammatory Bowel Disease) children with normal levels (<70 mcg/g) and high levels (>70 mcg/g) of fecal calprotectin.

The mean of KIDMED score was different between IBD children with normal levels (<70 mcg/g) and high levels (>70 mcg/g) of calprotectin. In particular, IBD patients with low values of calprotectin showed a significant higher KIDMED score mean (5.82 ± 2.35 vs. 4.85 ± 2.16; *p* = 0.027) (Figure 2). We did not find other significant differences in terms of KIDMED score and other parameters of inflammation (ferritin, iron, CRP, ESR, PUCAI/PCDAI). Moreover, we did not find any significant correlation between macro and micronutrients intake and these inflammation parameters.

### 3.4. Comparison between IBD Patients and RDA

There were significant differences in the reported intakes of the patients with IBD and the reference intakes from RDA for several dietary components. Compared to RDA, the intake of patients with IBD was significantly lower in kilocalories, fibres, folic acid, iron (only in females between 11–17 age) and some micronutrients (calcium, potassium, zinc). On the contrary it was significantly higher in protein (*p* < 0.001). Regarding vitamin intake, we found in IBD patients a significant lower intake in Vitamin D (*p* < 0.001), Vitamin A (only in males between 11–17 age, *p* < 0.001); Vitamin C (only in males between 15–17 age, *p* < 0.001), Vitamin E (only in females between 15–17 age, *p* = 0.002 and in males between 11–14 age, *p* = 0.0028). (Table 4)

## 4. Discussion

The present single-center study describes the dietary patterns and food intake at one-time point in pediatric IBD patients in remission, compared to a representative sample of healthy children. Moreover, we assess for the first time in IBD children adherence to the Mediterranean Diet and its correlation with inflammation.

As previously reported, our study confirms that children and adolescents with IBD compared to the RDA have significant lower intake of kilocalories and carbohydrates, as well as a deficiency of several micronutrients and fibers. Instead, the protein intake exceeded the RDA in accordance with a previous study from Hartman et al. [12]. Interestingly, IBD patients in remission compared to healthy controls showed a significantly higher intake of several macro and micronutrients. These differences can be explained because RDA presents the recommended nutrients intakes according to our best scientific knowledge. On the contrary, our control group reports the ‘‘real life” food intake habits for the general pediatric population. Therefore, given that RDA should continue to be our reference tool when giving dietary advice, it is suggested that we should also examine the intake information of the studied group in comparison to local data, as food habits may be quite different.

Traditionally, children with IBD have been described as malnourished and underweight. However, in two recent pediatric studies, it has been demonstrated that a significant proportion of IBD patients had a BMI consistent with being overweight or were at risk for being overweight [19,20]. Since the earliest descriptions of IBD, a greater number of medications have become available, and so it may be diagnosed earlier in the course of the disease. Therefore, malnourishment may no longer be a common manifestation of treated pediatric IBD. Indeed, malnutrition and weight loss are frequently observed in IBD patients at diagnosis as a result of many factors such as reduced dietary intake, malabsorption, diarrhea, and oxidative stress [21,22]. These phenomena are well known in patients with active disease, resulting from both inflammation and mucosal damage, however little is known regarding dietary habits in IBD pediatric patients during remission.

There is only one study [23] analyzing diet of CD adult patients in remission, which reports that macronutrients intake were covered by food intake, and only micronutrient deficiencies were present. On the contrary, a more recent pediatric study from Dierden at al. found that the diet of children with IBD differs from the general pediatric population, with lower energy intake [24]. More specifically, protein intake did not differ, while fat intake was higher in pediatric IBD and carbohydrate intake was lower. On the contrary, we found that there was a higher kilocalorie intake in patients with IBD compared to healthy controls, and an increase in carbohydrate and protein intake, with no significant difference in fat intake. These different results could be due the fact that part of the patients included in the study from Dierden et al. had active disease, which is known to be one of the most important causes of malnutrition [25].

Similar to Dierden et al., our study found that despite the lower energy intake in our patients, the BMI Z-scores were not impaired overall. Finally, in the study from Dierden et al. dietary intake was assessed by using the food frequency questionnaire (FFQ), which tends to overestimate intakes in adult validation studies [26,27,28]. On the contrary, the 3-day food record, used in our study, is considered to be the most accurate and precise method for dietary assessment in clinical practice [27].

The 3-day food record is the most time-consuming method for dietary assessment to administer: children and their parents had to be instructed in its use and then debriefed. Nonetheless, it offers the highest degree of accuracy of reporting with the lowest proportion of missing foods and phantom foods [29].

Interestingly, when we stratified patients according to disease subtype, we found that in CD patients there was a higher protein intake, as well as better intake of some micronutrients such as vitamin D and zinc. It could be speculated that CD patients manifest a more pronounced trust in healthy nutrition and greater engagement in controlling their diet given the importance of nutrition, in particular enteral nutrition, as a treatment for the disease.

Finally, despite a low consumption in fibers, as previously reported [10,12,13,14,15,16,17,18,19,20,21,22,23,24] and the low-intermediate adherence to MD reported in our IBD patients, we found a significant inverse correlation between adherence to MD and fecal calprotectin. Associations between nutrients and inflammation have been established [30]. Previous research in non-IBD populations has demonstrated the positive impact of dietary behaviors on inflammation and inflammatory markers, including C reactive protein [31,32].

There is a large body of evidence proving that Mediterranean dietary patterns regulate inflammation in chronic disease [33,34,35] and in individuals with IBD; there is some evidence that adherence to MD is associated with lower risk of developing IBD [36,37] and decreased inflammation [38,39]. The results of our study stimulate further interest in the relationships between dietary patterns, nutrition adequacy, and disease pathogenesis in IBD. This relationship between dietary behaviors and inflammatory markers in children with IBD is particularly important since it could have implications on health outcomes associated with chronic inflammation and active disease, including malnutrition, growth failure, osteoporosis, lower bone mineral density, and colorectal cancer [40,41,42].

The strength of this study is that we assessed the diet in a large cohort of pediatric IBD patients under medical guidance with a reliable tool and compared this to a matched cohort of children from the general population. Furthermore, to avoid bias, we excluded patients who had active disease or a had different diet related to therapy or bowel resection.

## 5. Conclusions

In conclusion, our study provides data on the nutritional status and dietary intake of pediatric patients with IBD in remission; however, it reveals the complexity of the relationship between nutrient intake and disease activity in children with IBD. Furthermore, our study suggests the need for evaluating the intake of IBD children compared to the healthy population, as it provides different information compared toRDA.

Finally, our patients reported food choices with restricted nutrient intake compared to a Mediterranean dietary pattern. Given the anti-inflammatory properties shown by MD, our results suggest that children with IBD should be screened for nutrient deficiencies and inadequacy of dietary patterns. However, further investigation is needed to test the efficacy of clinical outcomes of nutrition interventions promoting the MD pattern.

## Figures and Tables

**Table 1:** Characteristics of 125 inflammatory bowel disease (IBD) patients enrolled and 125 healthy controls (HCs).

Characteristics	IBD	HCs	*p*
	*n* = 125	*n* = 125	
Age in years, Median (range)	15 (5–17)	14 (5–17)	0.312
Gender, n (%)			1
Male	68 (54.4)	67 (53.6)	
Female	57 (45.6)	58 (46.4)	
Disease, n (%)		NA	
CD	53 (42.4)		
UC	72 (57.6)		
PCDAI/PUCAI (median, range)	0 (0–15)	NA	
Paris classification at diagnosis, n (%)		NA	
CD			
Ileum only (L1)			
Colon only (L2)	13 (24.5)		
Ileum and colon (L3)	5 (9.4)		
Upper gastrointestinal tract (L4)	31 (58.5)		
	3 (5.6)		
UC			
Ulcerative proctitis (E1)			
Left-sided UC (E2)	9(12.5)		
Extensive UC (E3)	12(16.6)		
Pancolitis (E4)	13(18)		
	38(52.7)		
Therapy, n (%)		NA	
5-ASA	60(48)		
Immunomodulators (AZA, MTX)	47(37.6)		
Anti-TNFα (IFX, ADA)	14(11.2)		
Vedolizumab	1(0.8)		
None	3(2.4)		
Weight zscores, mean (±SEM)	0.15 ± 0.11	0.15 ± 0.09	0.9865
Height z scores, mean (±SEM)	−0.12 ± 0.10	−0.14 ± 0.12	0.9344
BMI z scores, mean (±SEM)	0.18 ± 0.11	0.35 ± 0.09	0.2427

Legend; IBD: Inflammatory Bowel Disease; CD: Crohn’s disease; UC: Ulcerative colitis; HCs: Healthy controls; PCDAI: Pediatric Crohn’s Disease Activity Index; PUCAI: Pediatric Ulcerative Colitis Activity Index; 5-ASA: 5-aminosalicyl-acid, AZA: Azathioprine; MTX: Methotrexate; Anti-TNFα: anti tumor necrosis factor α; IFX: Infliximab; ADA: Adalimumab; BMI: Body Mass Index; NA: not applicable.

**Table 2:** Energy Intake, Macro and micronutrients between children with IBD and Healty Controls (HCs).

Characteristics	IBD	HCs	*p*
Energy Intake (kcal/day), mean (±SEM)	1825.25 ± 46.12	1673.15 ± 38.74	0.0122
Macronutrients, mean (±SEM)			
Total protein (g/day)	72.52 ± 2.17	66.09 ± 1.82	0.024
Lipids (g/day)	79.83 ± 2.05	76.22 ± 2.37	0.25
Carbohydrates (g/day)	215.00 ± 7.29	193.17 ± 5.19	0.0154
Starch (g/day)	137.47 ± 5.14	127.48 ± 3.95	0.1248
Oligosaccharides (g/day)	42.12 ± 2.14	42.23 ± 1.61	0.9692
Cholesterol (mg/day)	205.62 ± 10.41	194.03 ± 10.40	0.4317
Saturated fatty acids (g/day)	20.5 ± 0.77	20.34 ± 0.84	0.909
Poly-unsaturated fatty acids (g/day)	9.36 ± 0.38	8.09 ± 0.36	0.0167
Fiber (g/die)	13.61 ± 0.55	13.46 ± 0.43	0.8324
Micronutrients, mean (±SEM)			
Calcium (mg/day)	446.05 ± 20.43	414.71 ± 17.05	0.24
Sodium (mg/day)	1633.40 ± 105.47	1390.46 ± 88.53	0.789
Potassium (mg/day)	2012.89 ± 58.56	1882.94 ± 51.02	0.0956
Phosphorus (mg/day)	1041,65 ± 30.78	936.69 ± 26.40	0.0102
Iron (mg/day)	9.4 ± 0.31	8.65 ± 0.25	0.067
Zinc (mg/day)	9.10 ± 0.32	7.94 ± 0.26	0.0058
Folic Acid (mcg/dy)	183.15 ± 8.27	186.21 ± 8.04	0.791
Niacin (mg/day)	15.8 ± 0.67	14.24 ± 0.57	0.07
Riboflavin (mg/day)	1.67 ± 0.18	1.27 ± 0.12	0.052
Thiamine (mg/day)	1.12 ± 0.04	0.97 ± 0.03	0.0041
Vitamin A (mcg/day)	438.5 ± 31.97	451.15 ± 33.84	0.787
Vitamin B6(mg/day)	1.5 ± 0.06	1.4 ± 0.05	0.041
Vitamin C (mg/day)	71.89 ± 6.05	66.75 ± 5.80	0.5398
Vitamin D (mcg/day)	6.49 ± 1.39	2.78 ± 0.30	0.132
Vitamin E (mg/day)	11.99 ± 0.30	11.28 ± 0.28	0.041
KIDMED score, n (%)			0.7664
Optimal	20 (16.1%)	17 (15.6%)	
Intermediate	71 (56.9%)	67 (61.5%)	
Low	33 (26.6%)	25 (22.9%)	

Legend; IBD: Inflammatory Bowel Disease; HCs: Healthy controls; KIDMED: Mediterranean Diet Quality Index for Children and Adolescents.

**Table 3:** Comparison between Crohn’s disease and Ulcerative colitis patients regarding anthropometric parameters, KIDMED, macro and micronutrient values.

Characteristics	CD	UC	*p*
	(*n* = 53)	(*n* = 72)	
Sex			0.0037
Male n, (%)	37 (69.8%)	31 (43.1%)	
Femalen, (%)	16 (30.2%)	41 (56.9%)	
Age mean (sd)	14.77 ± 0.30	14.17 ± 0.37	0.2263
Weight z-score, mean (±SEM)	0.07 ± 0.13	0.21 ± 0.16	0.276
Height z-score, mean (±SEM)	−0.21 ± 0.14	-0.06 ± 0.15	0.342
BMI z-score, mean (±SEM)	0.11 ± 0.15	0.23 ± 0.16	0.367
KIDMED score, n (%)			0.5183
Low	15 (28.3%)	18 (25.4%)	
Intermediate	32 (60.4%)	39 (54.9%)	
Optimal	6 (11.3%)	14 (19.7%)	
KIDMED, mean (±SEM)	4.87 ± 0.30	5.35 ± 0.27	0.384
Dietary intake, mean (±SEM)			
Energy Intake (kcal/day)	1942.14 ± 65.80	1739.20 ± 62.20	0.225
Protein (g/day)	80.10 ± 3.20	66.93 ± 2.78	0.047
Lipids (g/day)	83.42 ± 3.29	77.20 ± 2.58	0.428
Carbohydrates (g/day)	230.60 ± 10.29	203.52 ± 9.98	0.283
Starch (g/day)	144.57 ± 6.60	132.24 ± 7.46	0.702
Oligosaccharides (g/day)	40.94 ± 3.46	42.99 ± 2.72	0.534
Fiber (g/die)	13.70 ± 0.81	13.54 ± 0.75	0.658
Cholesterol (mg/day)	221.11 ± 12.22	194.21 ± 15.62	0.436
Saturated fatty acids (g/day)	21.54 ± 1.13	19.68 ± 1.03	0.599
Poly-unsaturated fatty acids (g/day)	10.03 ± 0.63	8.86 ± 0.47	0.602
Calcium (mg/day)	465.91 ± 32.61	431.42 ± 26.16	0.677
Sodium (mg/day)	1744.70 ± 164.15	1551.47 ± 137.77	0.733
Potassium (mg/day)	2109.42 ± 64.70	1941.84 ± 79.77	0.584
Phosphorus (mg/day)	1108.34 ± 47.66	992.56 ± 39.89	0.47
Iron (mg/day)	10.52 ± 0.49	8.54 ± 0.37	0.023
Zinc (mg/day)	9.83 ± 0.54	8.56 ± 0.38	0.37
Folic Acid (mcg/day)	194.42 ± 12.35	174.86 ± 11.10	0.554
Niacin (mg/day)	17.80 ± 1.04	14.38 ± 0.84	0.103
Riboflavin (mg/day)	1.83 ± 0.25	1.54 ± 0.24	0.834
Thiamine (mg/day)	1.23 ± 0.07	1.04 ± 0.05	0.15
Vitamin A (mcg/day)	439.99 ± 54.74	437.42 ± 38.45	0.949
Vitamin B6 (mg/day)	1.72 ± 0.11	1.38 ± 0.07	0.084
Vitamin C (mg/day)	73.89 ± 9.97	70.42 ± 7.57	0.906
Vitamin D (mcg/day)	10.80 ± 2.96	3.32 ± 0.92	0.044
Vitamin E (mg/day)	12.55 ± 0.52	11.57 ± 0.34	0.243

Legend; IBD: Inflammatory Bowel Disease; CD: Crohn’s disease; UC: Ulcerative colitis; HCs: Healthy controls; KIDMED: Mediterranean Diet Quality Index for Children and Adolescents.

**Table 4:** Reported 3 days food intake compared with Italian RDA.

Nutrient	Sex	Age (y)	Median (Q1;Q2)	Reference Value	*p*-Value
Energy intake (kcal/day)	M	13	2061 (1562;2106)	2780	0.031
	M	14	2043 (1550;2200)	2960	0.002
	M	15	1939 (1469;2317)	3110	0.004
	M	16	2005 (1700;2598)	3210	<0.001
	M	17	1870 (1602;2172)	3260	<0.001
	F	13	1621 (1120;1731)	2780	0.062
	F	14	1724 (1137;1957)	2960	0.004
	F	15	1470 (1216;1681)	3110	0.008
	F	16	1321 (1289;1484)	3210	0.004
	F	17	1645 (1430;1779)	3260	<0.001
			Mean (±SEM)		
Lipids (%kcal)	/	/	39.75 (±6.52)	40	0.670
Carbohydrates (%kcal)	/	/	46.49 (±7.77)	52.5	<0.001
Fiber (g/day)	/	/	7.56 (±3.19)	8.4	0.004
Proteins (g/day)	M	11–14	84.67 (±26.94)	48	<0.001
	M	15–17	82.99 (±25.48)	62	<0.001
	F	11–14	62.09 (±17.57)	48	0.003
	F	15–17	60.18 (±16.51)	50	0.001
Calcium (mg/day)	M	11–17	480.66 (±225.74)	1300	<0.001
	F	11–14	428.38 (±152.07)	1300	<0.001
	F	15–17	375.25 (±214.89)	1200	<0.001
Sodium (mg/day)	M	11–17	1762.59 (±1243.83)	1500	0.104
	F	11–17	1390.91 (±1023.79)	1500	0.450
Potassium (mg/day)	M	11–17	2217.43 (±617.67)	3900	<0.001
	F	11–17	1804.27 (±638.41)	3900	<0.001
Phosphorus (mg/day)	M	11–17	1201.52 (±367.08)	1250	0.306
	F	11–17	872.99 (±233.07)	1250	<0.001
Iron (mg/day)	M	11–14	10.19 (±3.91)	10	0.829
	M	15–17	34.68 (±151.22)	13	0.364
	F	11–17	7.96 (±2.66)	18	<0.001
Zinc (mg/day)	M	11–17	10.45 (±3.65)	12	0.002
	F	11–17	7.59 (±2.90)	9	0.001
Folic Acid (mcg/day)	M	11–14	211.58 (±114.62)	350	<0.001
	M	15–17	194.69 (±81.68)	400	<0.001
	F	11–14	192.03 (±90.83)	350	<0.001
	F	15–17	148.61 (±70.33)	400	<0.001
Niacin (mg/day)	M	11–14	18.54 (±8.48)	17	0.426
	M	15–17	23.30 (±30.61)	18	0.274
	F	11–14	14.26 (±5.50)	17	0.044
	F	15–17	12.64 (±5.33)	18	<0.001
Riboflavin (mg/day)	M	11–14	1.52 (±0.69)	1.3	0.167
	M	15–17	2.26 (±2.77)	1.6	0.134
	F	11–14	1.30 (±1.21)	1.2	0.733
	F	15–17	1.21 (±1.40)	1.3	0.731
Thiamine (mg/day)	M	11–14	1.17 (±0.37)	1.1	0.424
	M	15–17	1.32 (±0.54)	1.2	0.177
	F	11–14	1.04 (±0.33)	1	0.618
	F	15–17	0.92 (±0.36)	1.1	0.007
Vitamin A (mcg/day)	M	11–14	432.75 (±268.55)	600	0.012
	M	15–17	429.08 (±369.89)	700	<0.001
	F	11–17	531 (±838.91)	600	0.561
Vitamin B6 (mg/day)	M	11–14	1.78 (±0.87)	1.2	0.008
	M	15–17	11.42 (±61.54)	1.3	0.299
	F	11–14	1.32 (±0.42)	1.2	0.218
	F	15–17	1.20 (±0.43)	1.3	0.210
Vitamin C (mg/day)	M	11–14	87.53 (±97.42)	90	0.911
	M	15–17	62.09 (±47.79)	105	<0.001
	F	11–14	88.17 (±83.41)	80	0.674
	F	15–17	59.79 (±54.11)	85	0.013
Vitamin D (mcg/day)	/	/	6.49 (±15.59)	15	<0.001
Vitamin E (mg/day)	M	11–14	12.90 (±3.59)	11	0.028
	M	15–17	12.19 (±3.40)	13	0.134
	F	11–14	11.96 (±2.73)	11	0.143
	F	15–17	10.83 (±2.00)	12	0.002

Legend; RDA: Recommended Dietary Allowance; M: male; F: female.

## Supplementary Materials

The following are available online at https://www.mdpi.com/2072-6643/12/10/3206/s1, Table S1. Energy Intake, Macro and micronutrients in children with IBD and Healty Controls (HCs). Table S2. Energy Intake, Macro and micronutrients in children with Crohn’s disease and Ulcerative colitis.

## Abbreviations

IBD	Inflammatory Bowel Disease
CD	Crohn’s disease
UC	Ulcerative colitis
HCs	Healthy controls
PCDAI	paediatric Crohn’s disease activity index
PUCAI	Pediatric Ulcerative Colitis Activity Index
MD	Mediterranean Diet
RDA	Recommended Daily Allowances
ESPGHAN	European Society for Paediatric Gastroenterology, Hepatology and Nutrition
