# Supplementary material for: Effectiveness of Mediterranean Diet’s Adherence in Children with Inflammatory Bowel Diseases
Source: Nutrients. 2020 Oct 20;12(10):3206. doi: 10.3390/nu12103206 (PMC7589768; doi:10.3390/nu12103206)
Supplement: Supplementary file 1 [file nutrients-12-03206-s001.pdf]

Table S1. Energy Intake, Macro and micronutrients in children with IBD and Healty Controls (HCs).

**Characteristics**	**IBD**		**HCs**	
Energy Intake , mean	1825.25 kcal/day		1673.15 kcal/day	
Macro nutrients, mean				
Total protein	72.52 g/day	40 g/1000kcal	66.09 g/day	39.44 g/1000kcal
Lipids	79.83 g/day	43.7 g/1000kcal	76.22 g/day	45.5 g/1000kcal
Carbohydrates	215.00 g/day	117.8 g/1000kcal	193.17 g/day	115.4 g/1000kcal
Starch	137.47 g/day	75.2 g/1000kcal	127.48 g/day	76.2 g/1000kcal
Oligosaccharides	42.12 g/day	23 g/1000kcal	42.23 g/day	25.2 g/1000kcal
Cholesterol	205.62 mg/day	112 mg/1000kcal	194.03 mg/day	116 mg/1000kcal
Saturated fatty acids	20.5 g/day	11.2 g/1000kcal	20.34 g/day	34 g/1000kcal
Poly-unsaturated fatty acids	9.36 g/day	5 g/1000kcal	8.09 g/day	4.83 g/1000kcal
Fiber	13.61 g/day	7.45 g/1000kcal	13.46 g/day	8 g/1000kcal
Micronutrients, mean				
Calcium	446.05 mg/day	244 mg/1000kcal	414.71 mg/day	247.8 mg/1000kcal
Sodium	1633.40 mg/day	894 mg/1000kcal	1390.46 mg/day	830.7 mg/1000kcal
Potassium	2012.89 mg/day	1102 mg/1000kcal	1882.94 mg/day	1125.3 mg/1000kcal
Phosphorus	1041,65 mg/day	1900 mg/1000kcal	936.69 mg/day	560 mg/1000kcal
Iron	9.4 mg/day	5.14 mg/1000kcal	8.65 mg/day	5.16 mg/1000kcal
Zinc	9.10 mg/day	5 mg/1000kcal	7.94 mg/day	4.74 mg/1000kcal
Folic Acid	183.15 mcg/day	100 mcg/1000kcal	186.21 mcg/day	111.3 mcg/1000kcal
Niacin	15.8 mg/day	8.65 mg/1000kcal	14.24 mg/day	8.51 mg/1000kcal
Riboflavin	1.67 mg/day	0.91 mg/1000kcal	1.27 mg/day	0.75 mg/1000kcal
Thiamine	1.12 mg/day	0.61 mg/1000kcal	0.97 mg/day	0.57 mg/1000kcal
Vitamin A	438.5 mcg/day	240 mcg/1000kcal	451.15 mcg/day	269.6 mcg/1000kcal
Vitamin B6	1.5 mg/day	0.82 mg/1000kcal	1.4 mg/day	0.83 mg/1000kcal
Vitamin C	71.89 mg/day	39.3 mg/1000kcal	66.75 mg/day	39.8 mg/1000kcal
Vitamin D	6.49 mcg/day	3.5 mcg/1000kcal	2.78 mcg/day	1.6 mcg/1000kcal
Vitamin E	11.99 mg/day	6.5 mg/1000kcal	11.28 mg/day	6.74 mg/1000kcal

Legend:IBD: Inflammatory Bowel Disease; HCs : Healthy controls.

Table S2. Energy Intake, Macro and micronutrients in children with Crohn's disease and Ulcerative colitis.

	**CD (*n*=53)**		**UC (*n*=72)**	
Dietary intake, mean				
Energy Intake	1942.14 kcal/day		1739.20 kcal/day	
Protein	80.10 g/day	41.2 g/1000kcal	66.93 g/day	38.4 g/1000kcal
Lipids	83.42 g/day	43 g/1000kcal	77.20 g/day	44.3 g/1000kcal
Carbohydrates	230.60 g/day	118.7 g/1000kcal	203.52 g/day	117 g/1000kcal

Starch	144.57 g/day	74.5 g/1000kcal	132.24 g/day	76 g/1000kcal
Oligosaccharides	40.94 g/day	21 g/1000kcal	42.99 g/day	24.7 g/1000kcal
Fiber	13.70 g/day	6.72 g/1000kcal	13.54 g/day	7.78 g/1000kcal
Cholesterol	221.11 mg/day	113.8 mg/1000kcal	194.21 mg/day	111.6 mg/1000kcal
Saturated fatty acids	21.54 g/day	11 g/1000kcal	19.68 g/day	11.3 g/1000kcal
Poly-unsaturated fatty acids	10.03 g/day	5.1 g/1000kcal	8.86 g/day	5 g/1000kcal
Calcium	465.91 mg/day	235.2 mg/1000kcal	431.42 mg/day	248 mg/1000kcal
Sodium	1744.70 mg/day	89.8 mg/1000kcal	1551.47 mg/day	892 mg/1000kcal
Potassium	2109.42 mg/day	1086 mg/1000kcal	1941.84 mg/day	1116 mg/1000kcal
Phosphorus	1108.34 mg/day	570.6 mg/1000kcal	992.56 mg/day	570.6 mg/1000kcal
Iron	10.52 mg/day	5.41 mg/1000kcal	8.54 mg/day	5 mg/1000kcal
Zinc	9.83 mg/day	5.06 mg/1000kcal	8.56 mg/day	5 mg/1000kcal
Folic Acid	194.42 mcg/day	100 mcg/1000kcal	174.86 mcg/day	100.5 mcg/1000kcal
Niacin	17.80 mg/day	9.16 mg/1000kcal	14.38 mg/day	8.26 mg/1000kcal
Riboflavin	1.83 mg/day	0.94 mg/1000kcal	1.54 mg/day	0.88 mg/1000kcal
Thiamine	1.23 mg/day	0.63 mg/1000kcal	1.04 mg/day	0.6 mg/1000kcal
Vitamin A	439.99 mcg/day	226.5 mcg/1000kcal	437.42 mcg/day	251.5 mcg/1000kcal
Vitamin B6	1.72 mg/day	0.88 mg/1000kcal	1.38 mg/day	0.8 mg/1000kcal
Vitamin C	73.89 mg/day	38.04 mg/1000kcal	70.42 mg/day	40.5 mg/1000kcal
Vitamin D	10.80 mcg/day	5.56 mcg/1000kcal	3.32 mcg/day	2 mcg/1000kcal
Vitamin E	12.55 mg/day	6.46 mg/1000kcal	11.57 mg/day	6.65 mg/1000kcal

Legend: IBD: Inflammatory Bowel Disease; CD: Crohn’s Disease; UC: Ulcerative colitis; HCs : Healthy controls
